# Healthcare access for refugee women with limited literacy: layers of disadvantage

**DOI:** 10.1186/s12939-017-0694-8

**Published:** 2017-11-10

**Authors:** Annette Floyd, Dikaios Sakellariou

**Affiliations:** 1 Schools of Health Sciences and Nursing, Langara College, Vancouver, Canada; 2 Cardiff University, School of Healthcare Sciences, Eastgate House, Newport Road 35-43, Cardiff, CF24 0AB UK

**Keywords:** Access to healthcare, Canada, Disadvantage, Refugees, Refugee and asylum seeker healthcare, Women’s health

## Abstract

**Background:**

Record numbers of people, across the world, are forced to be displaced because of conflict or other violations of their human rights, thus becoming refugees. Often, refugees not only have a higher burden of disease but also compromised access to healthcare, as they face many barriers, such as limited knowledge of the local language. However, there is very limited knowledge on the lived experiences of this population. Moreover, the strategies people might develop in their efforts to access healthcare have not been explored in depth, despite their value in establishing peer- support, community based programs.

**Methods:**

In this article, we present the findings of a study aiming to explore the lived experiences of accessing healthcare in the greater Vancouver area for recently-arrived, government-assisted refugee women, who were non-literate and non-English-speaking when they arrived in the country. We carried out sixteen semi-structured interviews with eight refugee women, guided by descriptive phenomenology.

**Results:**

The findings highlight the intersection of limited knowledge of the local language with low literacy, gender, and refugee status and how it impacts women’s access to healthcare, leading to added layers of disadvantage. We discuss three themes: (1) *Dependence*, often leading to compromised choice and lack of autonomy, (2) *Isolation*, manifesting as fear in navigating the healthcare system, rejection, or shame for a perceived inadequacy, and (3) *Resourcefulness* in finding ways to access healthcare.

**Discussion:**

We propose that a greater understanding of the intersections of gender, low literacy, and refugee status can guide healthcare workers and policy makers in improving services for this population. Furthermore, It is important to enable seldom-heard, hard to reach populations and facilitate their participation in research in order to understand how vectors of disadvantage intersect.

## Introduction

According to the United Nations Commissioner for Refugees [1], more than 65 million people worldwide have been displaced due to conflict, violence, persecution, or other violations of their human rights. Often, these people have a higher burden of disease, due to their exposure to physical and psychological trauma during the many stages of their journey from the country they fled to the host country and thus, they may have ongoing health needs requiring access to healthcare [2, 3]. Despite this, there is evidence that refugees’ access to healthcare is often compromised due to a variety of factors, including appropriateness of services, inflexibility of local systems, language barriers, and lack of transportation [2–5].

To date, only a few researchers have explored the experience of accessing healthcare from the perspective of the refugees themselves [6–8] and they all identified barriers, including transportation and language. Greater knowledge about the experiences of refugees accessing care is necessary to improve services. Furthermore, very little is known about how refugees cope with the problems they face in accessing healthcare. This is very important in order to enable an exploration of the strategies people develop, thus shifting the focus to their strengths and how these can be used to effect a positive change, through, for example, the establishment of community based peer-support programs.

In this article, we present the findings of a study exploring access to healthcare for refugee women with limited literacy in Canada, a population that often remains invisible in research. There is some evidence that low literacy contributes to poverty and reduced community engagement, both of which are associated with poorer health and quality of life [9]. Furthermore, there is evidence that women with limited literacy in low-income countries face greater problem accessing healthcare [10, 11]. Our aim in this article is to explore the lived experiences of accessing healthcare in the greater Vancouver area for recently-arrived, government-assisted refugee women of African origin, who were non-literate and non-English-speaking when they arrived in the country. We have a specific focus on the experience of both barriers to care and the facilitating factors that the women utilized in order to gain access to healthcare.

## Background

### Government-assisted refugees in Canada

Refugees are defined as people who have been forced to leave their country due to a well-founded fear of persecution or violence [12]. Canada offers permanent resettlement to approximately 7500 government-assisted refugees each year [13], and the numbers are increasing in response to the situation in Syria. These government-assisted refugees have their refugee status established abroad and are financially supported by the Canadian government.

In 2002, the Canadian government implemented the Immigration and Refugee Protection Act, which prioritised refugees in need of immediate protection. The result has been the arrival of more refugees with multiple barriers, including those with no knowledge of Canada’s official languages and low literacy levels in their first language [14]. Providers working with refugees have noted the difficulties that their clients have in navigating the healthcare system in Canada [15].

Most government-assisted refugees (GARs) coming to Canada from African countries have often spent several years in refugee camps after fleeing their country of origin. Upon arrival in Vancouver, they are provided temporary housing by a local settlement organisation for 2 weeks. They also receive a brief health assessment at a refugee clinic located in central Vancouver. They are able to continue to receive healthcare through this clinic for their first few years in the country. Within 2 weeks post-arrival, all GARs must have found housing in the free market, often in the cheaper outer suburbs. In moving away from central Vancouver, GARs face a long commute back to the refugee clinic to receive healthcare in their language of choice. Alternatively, they can access care in local clinics, where there is no obligation on the healthcare practitioner to provide language services.

### Barriers to accessing healthcare

There is growing evidence that immigrants and refugees have difficulties accessing healthcare in Canada [16, 17]. Gushulak et al. [18] note that recently arrived immigrants to Canada are twice as likely to have difficulties accessing healthcare than those who were born in the country. The utilisation of preventive services, screening for certain cancers, and mental health services is known to be lower in immigrant populations [19, 20]. Such disparities raise concerns about inequalities in access [21]. The barriers people face, pertain to communication [9, 22, 23], and low levels of literacy [24, 25]. Language barriers have been associated with delays in seeking care, reduced compliance and harmful health outcomes [26, 27]. Such barriers are increasingly implicated as a source of health disparities between immigrants and native-born populations [28, 29].

The literature suggests that refugee women are particularly affected and they might even have lower expectations from healthcare services than other women residing in Canada [30]. As minority women who may have language barriers, they may face ethnic or racial prejudice and sexist behaviour in addition to the stress of adjusting to a new society [31]. Work and family responsibilities and social isolation may make accessing and utilising healthcare particularly challenging for refugee women [32]. Furthermore, women refugees are more likely to lack English language skills or formal education than their male counterparts [33]. This is sometimes due to gender roles in their home countries, which may have centred around maintaining the household and raising children [34]. As the primary care-givers of children in the household and because of pregnancy and childbirth, women may have had a greater need to accessing healthcare since coming to Canada [33].

## Methods

We used qualitative methodology, informed by descriptive phenomenology, to enable an exploration of the lived experiences of the participants. Phenomenology is a shift away from physical nature and causality to the study of human consciousness, subjectivity and the meaning of actions [35]. It provides a useful lens to researchers aiming to explore, describe and analyse the meaning of the lived experience of the individual [36].

### Sampling strategy

We used a purposive sampling approach [37]. Initial recruitment focused on women from East African origins. Women from this region represent a sizable proportion of non-literate refugee arrivals in British Columbia, because many came from rural backgrounds and prolonged periods in refugee camps and thus had little access to education. This was expanded to include women from all of sub-Saharan Africa, due to challenges in obtaining an adequate sample size.

Inclusion criteria included:

- Government-assisted refugee women over the age of 18.
- Originally from a sub-Saharan African country.
- Arrived in Canada after January 1, 2009 and before December 31, 2012.
- Women who self-reported that they had never attended formal education and were unable to read or write, or who were assessed as being at level 1 or below, according to the Canadian Language Benchmarks.
- Women who self-reported that they could not speak English before arriving in Canada or who began English language classes at level 1 or below according to the Canadian Language Benchmarks.

Exclusion criteria included women who had been identified by a healthcare worker as having mental disabilities or mental health challenges that would impair their ability to participate in the study.

### Access to the study population

At the time of the study, the first author was employed as a nurse at the refugee clinic through which all GARs receive initial health screening, and often continue to receive primary care in the community. She was not, however, involved in offering care to the participants. In order to avoid any perception of coercion into participation, we used third party recruitment. All GARs who are resettled to the greater Vancouver area are provided settlement assistance for 1 year through Resettlement Assistance Program (RAP) counselors. The first author invited RAP counselors to identify current or former clients who met the inclusion criteria. If the client agreed, their contact information was passed to the researcher, who then arranged to call the client with an interpreter to explain the details of the study and answer any questions.

### Data collection

Data were collected through the use of semi-structured interviews. The first author conducted two interviews with each participant, with the aid of an interpreter fluent in the participant’s language of choice. The intention was to carry out in-depth interviews, but this was not always possible, as we discuss in the limitations section. The initial interview was an attempt to gain in-depth descriptions of the experiences of the women as they sought to access healthcare. The opening question asked participants to reflect on a time in Canada when they were concerned about their health or that of one of their children and they had to access healthcare. Follow up questions focused on collecting further information about their experiences, including information about how the participants felt, and what actions they took and why. A second interview was conducted after initial analysis of the first interview was completed. This second interview involved having the participant verify the researcher’s (first author) summary of the first interview, providing clarification, if needed, and expanding on any themes that the participant wanted to elaborate on. Basic demographic data were also collected.

As Merry et al. [38] advise, we tried to develop a data collection plan sensitive to the needs of this population. Interviews took place at either the participant’s home or at a local primary care clinic for refugees, depending on the individual’s preference. Transit passes and a childcare provider were made available, if needed, and a gift card for a local grocery store was offered to compensate for the participant’s time. With the aid of a language instructor who specialised in illiteracy, we developed an interview guide, so that prompts or questions were framed in a way that was accessible to a participant with limited literacy.

Each interview began with an informal conversation aimed at creating a relaxed atmosphere. The women were then asked to describe experiences when they had tried to access healthcare since arriving in Canada. Initial interviews lasted between 24 and 45 min, with the average duration between about 40 min. Second interviews were briefer (20–25 min), with most women agreeing with the summary presented to them and only a few expanding on the themes. All interviews were digitally recorded with consent from the participant. Field notes on the researcher’s initial thoughts and feelings about the interview were taken after each interview. In addition, a short de-briefing with the interpreter took place after each interview to clarify any details about the process.

### Data analysis

In keeping with the traditions of phenomenological research, data analysis focused on identifying themes contained within the descriptions given by the participants [36]. Through data analysis, we wanted to explore the essence of the experiences of the participants and to synthesise the essence into an integrated whole. Analysis was based on the process developed by Moustakas as an adaptation of Van Kaam’s methods [39]. The first step was to read each transcript in order to see what the main themes were, and then we re-read each transcript in a more focused way with the aim to produce an initial description of the themes. One of the themes was further broken down in subthemes, to enhance the specificity of the description of the participants’ experiences. This was not deemed to be necessary for all of the themes. The steps we followed are presented in Table 1.

**Table 1:** Analytical steps followed

Analytical steps
1. *Epoche*-which involves the setting aside of prejudgements, biases and preconceived ideas about things. This was done by having the researcher reflect on her assumptions about the research participants and their experiences and then attempting to bracket those assumptions away from the data that was generated through the interviews.
2. *Horizontalization-*which is achieved by listing all of the expressions within the data that are relevant to the experience. As each transcript was read, notes on possible emerging themes were written for each relevant expression.
3. *Reduction and elimination of irrelevant expressions.* This was achieved by reviewing each transcript and testing each expression as to whether it; a) contained a part of the experience that was necessary for understanding it and b) was able to be abstracted and labeled. Expressions that met both of these criteria were deemed to be invariant constituents of the experience.
4. *Developing themes and clustering the expressions.* Once themes began to emerge, expressions for each transcript were copied and pasted into a new Word document under headings of potential themes. Clusters were then read and additional themes developed.
5. *Validation of themes with the text.* Expressions and their corresponding themes were checked against the original transcripts to ensure their relevance to the participant’s experience.
6. *Individual textural and structural descriptions.* For each participant, an individual textural and structural description was written, which incorporated individual quotes from their interviews and themes that were derived.

In order to avoid being overtly influenced by the literature, which could impede our ability to discern the unique voices of the participants, we avoided carrying out an extensive literature review prior to the data collection, as advised by Moustakas [39]. Analysis followed an iterative process, so that each interview informed the next.

### Ethical considerations

We recognise that the study population was vulnerable due to their lack of understanding of English and also due to their gender and status as refugees. Given this, we took great care to ensure that potential participants were given information in their own language and adequate opportunity to ask questions and consult with family members, if they wished, before consenting to participate. We promoted an emphasis on the voluntary aspect of participation. In order to maintain confidentiality, participants were given numbers from the time of consent. Throughout this article, individual participants are not linked to any of their personal characteristics (such as age, marital status, or ethnicity) to lessen the possibility of participants’ identity being compromised. Prior to commencing the study, ethical approvals were granted by the London School of Hygiene and Tropical Medicine, UK (reference S10042/04/13) and the University of British Columbia, Canada (reference H13–00929). Permissions were also granted by the settlement organisation and local health authority.

## Results

Eight women who met the inclusion criteria agreed to participate in the study. Their ages ranged from 20 to approximately 38 years old (few of the women knew their exact age). One woman had only been in Canada 6 months, two for 2 years, and five for 3 years. Three of the women were Somali, two Liberian, two Eritrean, and one Ethiopian. Two of the women had husbands living with them in Canada, one had a husband who was still in Africa, and five were single. All but one of the women had at least one child. While each had been non-literate and did not speak English when they arrived in Canada, all but one had since learned basic English and enough literacy to write down simple numbers and to sign the consent form. In this section, we present the three main themes that emerged from the data: dependence, isolation, and resourcefulness. These themes describe participants’ lived experiences accessing healthcare of the participants, with a special focus on barriers to access and coping strategies. *Isolation* encompasses three subthemes, to add specificity to the experiences of the participants. In the quotations, participants are referred to as P (participant) and by the use of two numbers. The first number identifies the participant and the second the interview, so that P1–2 refers to a quotation by the first participant (in the order participants were recruited), in her second interview.

### Dependence

Language barriers, an inability to read directions or maps, and general unfamiliarity with an urban environment, meant that all of the participants were, at least initially, dependent on others to access healthcare in their new city. Neighbours, settlement workers, volunteers, family members, and even strangers were necessary for the women to gain access to the healthcare system.

The ability to know when appointments were was a challenge for the women, as they were unable to read an appointment slip or write the date of the appointment on a calendar. The refugee-specific clinics would call the women to remind them of their upcoming appointments, a practice that was greatly appreciated by the women. Some of the participants relied on their children to help them remember appointments, as one participant explained:

So I’ll remember, and also, I’ll talk to the children, so we’ll be reminded too about that. (P2–1).

Finding their way to the refugee clinic was a challenge for some participants, as it involved negotiating public transit, often for long distances and multiple buses. Initially, this was done with the help of volunteers, neighbours, or family members as the following quotation illustrated: “*There's a Somali guy who we know, so he's the one who brought us over*” (P1–1). Eventually, the women, either out of necessity or determination, would negotiate their way to the clinic on their own, relying on strangers along the way to assist them:

I keep showing it [the address] to the people on the street. ‘I’m looking for this place’. And they tell me. (P8–1).

As the refugee clinics were only open during regular business hours, the women also used walk-in fee-for-service clinics or hospitals near their homes when they or their children were ill. A sick child, for example, would result in calls to neighbours or community members that spoke their language. In one woman’s case, assistance was received from a neighbor’s child: “*A boy who's 10 years old took me there*” (P8–1)*.*

As the walk-in-clinics had no interpreter services the women often relied on neighbours, friends, or on their children to interpret for them. One single mother explains: “*My daughter talked to them, explained his sickness*” (P2–1)*.* In the case of community members or children interpreting, the conversation between the woman and her healthcare provider may be muted as both the woman and the interpreter may not feel comfortable with details relating to the woman’s health. The reliance on interpreters, often non-professional, placed a barrier between the women and their healthcare provider and the women were not always in full control of the interaction.

### Isolation

Language barriers, literacy challenges, and adaptation to their new environment left most of the women feeling isolated while accessing healthcare in Canada. This isolation was experienced in a number of ways: rejection, fear, and shame.

#### Rejection

Rejection may occur within the women’s own community as they ask for help in accessing healthcare. Newly arrived and needing healthcare for her child, one of the participants remembers that:

So I… first I talked, when my child was sick, I talked to the Somalis that I know. And… they didn’t do anything. (P7–1).

Though married, this woman had lost contact with her husband and thus arrived in Canada alone with her children. Single refugee women may be at particular risk of social isolation if cultural norms that govern a women’s role in society make it difficult for them to become part of larger refugee communities [32]. It may be that this woman’s status as a single mother isolated her within her own community and thus, made it particularly difficult for her to access resources.

Language barriers can lead to rejection from healthcare providers, who are under no obligation to provide interpreter services within their practice. One woman, who could not communicate effectively with a specialist, was simply turned away:

So the first visit, she went by herself, there was no way that they can communicate with the doctor and the specialist called the clinic, said, ‘We can’t communicate’, so there was nothing done at that visit. And she has to wait 7 months to get a new appointment. (P4–1, as translated by the interpreter).

One woman, who was not provided with interpreter services when her son underwent surgery, experienced a rejection of her role as the child’s mother when healthcare workers did not communicate with her. Instead, they spoke with the woman’s social worker (who did not speak the woman’s language) about the child’s condition:

The way I felt was that I was treated like not seeing me as a mother whose child had been taken away… They didn’t respect me or honour me being a mother or responsible for this child. (P3–1).

Others experienced racism within the healthcare setting. In the following case, the lay interpreter waited until after the visit was finished before she told the participant what had been said.

The doctor was an old man and when he see our ID [identification card], he said, “oh, from Sudan. Oh, Sudanese, they are the worst people”… I felt really bad. I’m a human like them. I have everything; the only thing that I cannot understand is language. (P8–2).

#### Fear

The inability to communicate with the society around them, both on the way to appointments and within the healthcare setting, resulted in a feeling of fear for some of the women. Challenges in negotiating their way to the hospital or clinic meant that for some of the women, calling the ambulance was seen as the better option than trying to get to the hospital by themselves. In some cases, the fear of getting lost on the way to an appointment would prevent participants from even attempting to get there, as one woman explained:

I hate Vancouver, because Vancouver is far away, and I’m afraid to get lost… if I have an appointment for long, like faraway places, I just don’t go. (P5–2).

Fear was also felt during their healthcare encounter when they were not able to fully understand what was going on. This usually occurred when they did not have access to an interpreter. Some of the women had developed enough English to have basic conversations, but when the appointment involved anything beyond the routine, the women often could not understand the details or ask questions. One of the women described a situation in which test results indicated that she needed to be seen by another doctor. Unable to fully understand why this was necessary, she was afraid:

They send me to another place. It makes me think different-like maybe something scary. That makes me scared. But I have to go because I want to know what’s happening. (P6–1).

Even during the experience of childbirth, women were sometimes without interpreters, unable to understand what was happening around them. Unable to communicate with the healthcare workers, one woman was left to remain in her bed, not knowing what decisions were being made about her care:

It was frightening not knowing the language…I had no idea what they [healthcare workers] were talking about or what they were going to do to me. (P1–1).

One woman described the experience of having to wait without an interpreter while her child underwent major surgery as her “*worst day*” since coming to Canada. As she watched the surgical team leave after the surgery was completed, there was no interpreter to inform her of her son’s condition:

All of them they went home and it was only me. I was waiting for him and they talked together and I thought that’s it, he won’t be alive, he’s not alive. (P3–1).

#### Shame

Within their new environment in Vancouver, the participants became very aware of their lack of formal education and how their life experience differed from most of the people around them. Their low literacy isolated them in the Canadian context, where 96% of the population has completed at least primary education [40]. Systems are developed with the assumption that people can read and understand English. Healthcare workers may not be familiar with working with non-literate clients. Several women expressed feelings of shame about their lack of education and language skills and the following quotation is indicative of how several of the participants felt:

I feel bad because I see people, like people write, people speak, people read. I cannot do that. I’d like to do that, but I cannot do that. I’m just like someone they take from the bush because my problem, my mom had no money, we never have chance to go to school. (P4–2).

Although many had made great strides in learning the language and some basic literacy, all continued to struggle. Their lack of perceived progress further added to their shame:

I felt bad because I thought that, still, I don’t know anything… I felt that still I don’t have enough. I cannot communicate. (P5–1).

### Resourcefulness

Despite their challenges, many of the women were determined to be courageous and to learn the skills that they needed to be able to access healthcare for themselves and their families. Some did not want to be a burden and thus after initial guidance from volunteers, they set out to negotiate their new environment on their own. One participant explains:

So when you ask people all the time, ‘help’, they will hate you by the end because- and I have children and my health and I cannot ask all the time people to get help and I don’t want to wait until I reach that point. I have to know, by myself, where to go and direction. I have to figure out by myself. (P7–2).

Unable to read signs or understand the announcements on public transportation, negotiating their way to their healthcare appointments is initially done through visual memory of particular landmarks at the location of the stops at which they must exit. As one woman explained: “*I tried to memorise the road and the bus*” (P1–1)*.*

Eventually, some of the women developed enough literacy to recognise the numbers of buses or differentiate between the two different train lines that ran into the city. Although they were unable to read it, having a piece of paper with the address of the clinic on it was a great asset to the women, as they could show it to strangers on the street and be pointed in the direction that they needed to go. When they attended clinics or hospitals without an interpreter, they tried to get by with hand gestures or would use their mobile phones to call friends who could translate for them.

While they recognised their lack of education as being unusual for women in Canada, a number of the participants were determined to move forward with their lives and to gain independence. While some referred to themselves as being “*stupid*” when they arrived in the country, others recognised that their challenges were a result of a lack of education and not a deficit within themselves. As one woman explained:

To be smart is very important because if I’m not smart and not knowing the language, I don’t know what to do. I could have stayed at home. But using my brain, this was important. (P6–1).

Many took pride in the advances that they had made in acquiring language and basic literacy. One woman spoke of how when she arrived in Canada she did not know her age and was unable to say when her birthday was. This led to great embarrassment as clerks in hospitals expressed shock at the idea that someone would not know how old they were. But after several years in language school, she was now able to answer the question, with great pride:

Sometimes I go to hospital. People say, ‘Do you know your birthday? Do you know what time you come at?’ When now people ask me, I say yes. I explain. (P8–1).

## Discussion

This study shows that the women initially faced significant challenges in accessing healthcare in greater Vancouver. Arriving in a society structured for a population with high levels of literacy and English language skills, the participants’ lack of these skills meant that they were initially unable to negotiate systems on their own, thus recognised as not self-sufficient. They depended on others for locating healthcare facilities and the directions to them. They also felt isolated, due to fear, shame, or rejection. This is consistent with previous research that indicated that language barriers and literacy have an adverse effect on healthcare access [17] and more widely on welfare support [41]. However, the participants also demonstrated resourcefulness, exemplified through the use of effective ways of coping.

In 1971, British family doctor Hart proposed the inverse care law, whereby the “*availability of good medical care tends to vary inversely with the need for it in the population served*” ([42].p. 405). It is unfortunate that our findings suggests this is still the case, several decades after the law was first proposed, for refugee women in Canada. Despite a requirement from Canada Health Act that all provincial healthcare programs be accessible [43], this is not always the case for refugee women living in Vancouver.

This study adds to debates regarding access to healthcare for refugee women. More specifically, it adds to a body of literature specifically on the impact of illiteracy and limited knowledge of the local language on accessing healthcare [16, 44]. The findings highlight the intersection of limited knowledge of the local language with illiteracy, gender, and refugee status and how it impacts women’s access to healthcare. Refugee women are more likely to have limited knowledge of the local language or limited literacy because of their gender [16, 34] and this in turn can have a negative impact on their access to healthcare, leading to dependence or isolation. Adding to this, their gender means that they most often take caring responsibilities for their children, thus increasing their need to access healthcare [32], leading to added layers of disadvantage, or, adapting from Manderson and Warren [45], cascades of disadvantage.

Settlement service resources were unable to provide for a more extensive orientation that may have benefitted these women and thus they needed to rely on community volunteers or their children to access services. This raised issues such as lack of choice, autonomy, and confidentiality. With no obligation to have services provided in their own language, the women were sometimes turned away from clinicians or left to receive services with only gestures as a means of communication.

Clinics that are specifically for refugees were able to offer comprehensive care for the women, due to their provision of interpreters and the length of time that was allowed for the visit. This is congruent with the findings of a systematic review showing that the use of specialist refugee clinics, with interpreters and bilingual healthcare workers, is positively associated with better access and better quality of care [46]. Walk-in general practice clinics may have provided suboptimal care as the women often resorted to improvised hand signals or used young children as interpreters, and their fee-for-service structure usually resulted in a short visit. Messias et al. [44] discuss how lack of effective communication between clients and healthcare providers can undermine trust and increase the likelihood that the patient will not have adequate follow-up.

The lack of adequate settlement resources and the failure of the greater Vancouver health authorities to provide health services that were geographically and linguistically accessible to these women, services in their community, indicates a continuation of the social injustice to which these women have been subject to throughout their lives. War forced them to flee to countries where they spent years or even decades in refugee camps or urban slums. Their status as refugees, poverty, and their household responsibilities may have prevented them from obtaining even basic education.

On arrival in Canada they entered a system that valued self-sufficiency and independence, often leading to feelings of shame, dependence, rejection, and even fear. Dyck and McLaren [47] argue that Canada’s neoliberal political climate positively values self-sufficient immigrants, but negatively values refugees. In a system where the neoliberal values of cost-effectiveness and productivity have entered not only the public but also the domestic domain, refugee women with limited literacy skills are valued negatively as a burden to the system and sometimes to their own families (e.g. to their children, who sometimes offer help with translation). According to Dossa [48] in Canada racial minorities are “constructed as foreigners, desirable for their labour only” (pp.155). Refugee women with limited education, whose skills do not immediately lend them to employability, become socially constructed as problematic, dependent, and unable to adjust to life in Canada [49]. Language barriers and relegation to minority-group status can further contribute to women’s isolation and sense of displacement [50].

A recent change in Canadian citizenship criteria requires that immigrants have documented proof of attaining at least Level 4 of the Canadian Language Benchmarks. Such a requirement may make it impossible for these women to attain citizenship in Canada. Under-valued and voiceless within Canadian society, non-literate refugee women may not be able to advocate for services as other groups may. Refugees are often framed as a burden to already overburdened social systems [51].

However, it is important to note that the women in this study did not passively accept their barriers to accessing healthcare, but instead they set out into their new environments to access the care that they or their children required, demonstrating resourcefulness. Navigating lengthy and complicated rides on public transportation, or large healthcare facilities, the women used memorisation, visual clues, and a process of buddying up [8], enlisting help from friends, children, and also from strangers to gain access to healthcare. Few studies on resettled refugee women make reference to the means by which they overcome the challenges they face. This corresponds to literature that noted that refugees’ resilience and skills are rarely highlighted in research [51, 52]. Baird’s [53] study on resettled Sudanese refugee women identified similar pride felt by the participants when describing their abilities to be more independent. Similarly, Toth [54] described courage, determination, and perseverance as the characteristics that helped immigrant and refugee women overcome barriers.

## Limitations

Time constraints and recruitment challenges resulted in a small sample size, which may not reflect the experiences of all refugee women. Working with interpreters resulted in a fragmented conversation with some of the participants. This, and some of the participants’ hesitancy in discussing the details of their experiences, made it difficult to generate rich and deep descriptions. While we believe it is important to explore the lived experiences of this population, a methodology more reliant on observation rather than interviews, such as ethnography, might have been more appropriate.

As sampling was done through third-party recruiters, it is unknown if certain groups were over or under-represented. It is possible that women who were more confident and acculturated were more likely to participate. The interviewer (first author) was also a nurse working with refugee women, and her specific positioning might have influenced the interpretation of the results, leading, perhaps to a greater focus on the barriers these women faced.

Research indicates that the gender of the interpreter can have an impact on data collection [55]. Therefore, attempts were made to use only female interpreters. In one case this was not possible, and this may have inhibited the participant from speaking openly about her experiences, especially those related to pregnancy and childbirth. Despite its limitations due to its challenging nature, this study offers some much needed information about the experiences of a population, refugee women, who often remains neglected in research.

## Conclusions

This study contributes to the body of knowledge on access to healthcare for refugees and more specifically for women with limited literacy. It is important to enable seldom heard, hard to reach populations and facilitate their participation in research (through the use of interpreters, increased sensitivity, and a detailed consideration of ethical issues) in order to understand how vectors of disadvantage intersect. While often gender or refugee status are explored as discrete categories, in this article we illustrated how different factors can relate with each other to produce the possibilities for exclusion or compromised access to healthcare for women refugees. The findings demonstrate that despite the challenges they faced, participants were able to gain some access through maximising the resources at their disposal. Alongside increased interpreter and settlement services for refugee women and support with transportation, the implementation of peer-support [56] and also a bottom-up approach, whereby refugee women would explain the strategies they have developed, might contribute to reducing current inequities.

## Abbreviations

GARs: Government Assisted Refugees
RAP: Resettlement Assistance Program

## References

[1] United Nations High Commissioner for Refugees UNHCR. UNHCR global trends: forced displacement 2016. UNHCR. 2016. http://www.unhcr.org/576408cd7 Accessed 10 Aug 2017.

[2] Brolan CE, Forman L, Dagron S, Hammonds R, Waris A, Latif L, Ruano AL (2017). The right to health of non-nationals and displaced persons in the sustainable development goals era: challenges for equity in universal health care. Int J Equity Health.

[3] Langlois EV, Haines A, Tomson G, Ghaffar A (2016). Refugees: towards better access to health-care services. Lancet.

[4] Hyde R (2016). Refugees need health cards, say German doctors. Lancet.

[5] Kousoulis AA, Ioakeim-Ioannidou M, Economopoulos KP (2016). Access to health for refugees in Greece: lessons in inequalities. Int J Equity Health.

[6] Adair R, Nwaneri MO, Barnes N (1999). Health care access for Somali refugees: views of patients, doctors, nurses. Am J Health Behav.

[7] Asanin J, Wilson K (2008). “I spent nine years looking for a doctor”: exploring access to health care among immigrants in Mississauga, Ontario, Canada. Soc Sci Med.

[8] Woodgate RL, Busolo DS, Crockett M, Dean RA, Amaladas MR, Plourde PJ (2017). A qualitative study on African immigrant and refugee families’ experiences of accessing primary health care services in Manitoba, Canada: it’s not easy!. Int J Equity Health.

[9] Poureslami I, Rootman I, Doyle-Waters MM, Nimmon L, FitzGerald JM (2011). Health literacy, language, and ethnicity-related factors in newcomer asthma patients to Canada: a qualitative study. J Immigr Minor Health.

[10] Lam Y, Broaddus ET, Surkan PJ (2013). Literacy and healthcare-seeking among women with low educational attainment: analysis of cross-sectional data from the 2011 Nepal demographic and health survey. Int J Equity Health.

[11] LeVine RA, LeVine SE, Rowe ML, Schnell-Anzola B (2004). Maternal literacy and health behavior: a Nepalese case study. Soc Sci Med.

[12] United Nations High Commissioner for Refugees UNHCR. The State of the World’s Refugees. UNHCR. 1993. http://www.unhcr.org/4a4c6da96.html. Accessed 10 Aug 2017.

[13] United Nations High Commissioner for Refugees UNHCR. Frequently Asked Questions About Resettlement. UNHCR. 2012. http://www.unhcr.org/4ac0873d6.html. Accessed 10 Aug 2017.

[14] Hiebert D, Sherrell K. The Intergration and Inclusion of Newcomers in British Columbia Metropolis. Metropolis British Columbia. 2009. http://mbc.metropolis.net/assets/uploads/files/wp/2009/WP09-11.pdf. Accessed 5 Aug 2017.

[15] Folinsbee W., Quigley A. & Gregoire H. Cross-national Consultations on Health and Learning: Final Report on Adults with Literacy Challenges and Adult Immigrants and Refugees. Canadian Council on Learning. 2007. https://www.google.co.uk/url?sa=t&rct=j&q=&esrc=s&source=web&cd=1&ved=0ahUKEwivptTp_fDVAhVKKMAKHejBC-UQFggmMAA&url=http%3A%2F%2Fen.copian.ca%2Flibrary%2Fresearch%2Fhlkc%2Flit_imm_ref_sum_en%2Flit_imm_ref_sum_en.pdf&usg=AFQjCNGA9eq3uLDSi_Nz6HQVLYooxhWRvQ Accessed 5 Aug 2017.

[16] Lebrun LA (2012). Effects of length of stay and language proficiency on health care experiences among immigrants in Canada and the United States. Soc Sci Med.

[17] Stewart MJ, Neufeld A, Harrison MJ, Spitzer D, Hughes K, Makwarimba E (2006). Immigrant women family caregivers in Canada: implications for policies and programmes in health and social sectors. Health Soc Care Community..

[18] Gushulak BD, Pottie K, Roberts JH, Torres S, DesMeules M (2011). Migration and health in Canada: health in the global village. CMAJ.

[19] Hyman I (2004). Setting the stage: reviewing current knowledge on the health of Canadian immigrants: what is the evidence and where are the gaps?. CJPH.

[20] Latif E (2010). Recent immigrants and the use of cervical cancer screening test in Canada. J Immigr Minor Health.

[21] Chen AW, Kazanjian A, Wong H (2008). Determinants of mental health consultations among recent Chinese immigrants in British Columbia, Canada: implications for mental health risk and access to services. J Immigr Minor Health.

[22] Boateng L, Nicolaou M, Dijkshoorn H, Stronks K, Agyemang C (2012). An exploration of the enablers and barriers in access to the Dutch healthcare system among Ghanaians in Amsterdam. BMC Health Serv Res.

[23] Dastjerdi M, Olson K, Ogilvie L (2012). A study of Iranian immigrants’ experiences of accessing Canadian health care services: a grounded theory. Int J Equity Health.

[24] Sheikh-Mohammed M, MacIntyre CR, Wood NJ, Leask J, Isaacs D (2006). Barriers to access to health care for newly resettled sub-Saharan refugees in Australia. Med J Aust.

[25] Simich L. Health Literacy and Immigrant Populatons. Public Health Agency of Canada. 2009. health_literacy_policy_brief_jun15_e.pdf Accessed 10 Aug 2017.

[26] Government of Canada. Responsiveness of the Canadian Health Care System Towards Newcomers. Government of Canada. 2002. http://publications.gc.ca/site/eng/236833/publication.html Accessed 5 Aug 2017.

[27] Ng E, Pottie K, Spitzer D (2011). Official language proficiency and self-reported health among immigrants to Canada. Health Rep.

[28] Fennelly K (2006). Listening to the experts: provider recommendations on the health needs of immigrants and refugees. J Cult Divers.

[29] Lebrun LA, Dubay LC (2010). Access to primary and preventive care among foreign-born adults in Canada and the United States. Health Serv Res.

[30] Setia MS, Quesnel-Vallee A, Abrahamowicz M, Tousignant P, Lynch J (2011). Access to health-care in Canadian immigrants: a longitudinal study of the National Population Health Survey. Health Soc Care Community..

[31] Pittaway E, Bartolomei L. Refugees, race, and gender: The multiple discrimination against refugee women. Refuge. 2001;19(6):21–32.

[32] Vissandjée B, Weinfeld M, Dupéré S, Abdool S (2001). Sex, gender, ethnicity, and access to health care services: research and policy challenges for immigrant women in Canada. JIMI.

[33] Deacon Z, Sullivan C (2009). Responding to the complex and gendered needs of refugee women. Affilia.

[34] Israelite NK, Herman A, Alim FA (1999). Waiting for" Sharciga": resettlement and the roles of Somali refugee women. Can Woman Stud..

[35] Giorgi A (2005). The phenomenological movement and research in the human sciences. Nurs Sci Q.

[36] Van Manen M. Researching lived experience: Human science for an action sensitive pedagogy. Abingdon: Routledge; 2016.

[37] Green J. Principles of social research. Berkshire, England: Open University Press; 2008.

[38] Merry L, Clausen C, Gagnon AJ, Carnevale F, Jeannotte J, Saucier JF, Oxman-Martinez J (2011). Improving qualitative interviews with newly arrived migrant women. Qual Health Res.

[39] Moustakas C. Phenomenological research methods: Sage; 1994.

[40] World Bank. World Development Indicators: Education Completion and Outcomes. World Bank, 1996. http://wdi.worldbank.org/table/2.13. Accessed 10 Aug 2017.

[41] Team V, Markovic M, Manderson L (2007). Family caregivers: Russian-speaking Australian women's access to welfare support. Health Soc Care Community..

[42] Hart JT (1971). The inverse care law. Lancet.

[43] Government of Canada. Canada Health Act. Government of Canada. 2012. http://laws-lois.justice.gc.ca/eng/acts/C-6/page-2.html#h-4. Accesed 5 Aug 2017.

[44] Messias DK, McDowell L, Estrada RD (2009). Language interpreting as social justice work: perspectives of formal and informal healthcare interpreters. Adv Nurs Sci.

[45] Manderson L, Warren N (2016). “Just one thing after another”: recursive cascades and chronic conditions. Med Anthropol Q.

[46] Joshi C, Russell G, Cheng IH, Kay M, Pottie K, Alston M, Smith M, Chan B, Vasi S, Lo W, Wahidi SS (2013). A narrative synthesis of the impact of primary health care delivery models for refugees in resettlement countries on access, quality and coordination. Int J Equity Health.

[47] Dyck I, McLaren AT (2004). Telling it like it is? Constructing accounts of settlement with immigrant and refugee women in Canada. Gend Place Cult.

[48] Dossa P. Racialized bodies, disabling worlds: Storied lives of immigrant Muslim women. Toronto: University of Toronto Press; 2009.

[49] Mohamed HS (1999). Resistance strategies: Somali women's struggles to reconstruct their lives in Canada. Can Woman Stud.

[50] Berman H, Girón ER, Marroquín AP (2006). A narrative study of refugee women who have experienced violence in the context of war. CJNR.

[51] Grove NJ, Zwi AB (2006). Our health and theirs: forced migration, othering, and public health. Soc Sci Med.

[52] Beiser M. The health of immigrants and refugees in Canada. Can J Public Health. 2005:S30–44. 10.1007/BF03403701 PMC6976041 16078554

[53] Baird MB (2009). Resettlement transition experiences among Sudanese refugee women. Unpublished PhD thesis.

[54] Toth J (2003). Resilience: the experience of immigrant and refugee women. Unpblished master of nursing thesis.

[55] Roussos S, Mueller MR, Hill L, Salas N, Hovell M, Kronenfeld JJ (2010). Villarreal V. Some considerations regarding gender when a healthcare interpreter is helping providers and their limited English proficient patients. The impact of demographics on health and health care: race, ethnicity and other social factors.

[56] Stewart M, Simich L, Shizha E, Makumbe K, Makwarimba E (2012). Supporting African refugees in Canada: insights from a support intervention. Health Soc Care Community.

